# Cornel Iridoid Glycoside Inhibits Tau Hyperphosphorylation via Regulating Cross-Talk Between GSK-3β and PP2A Signaling

**DOI:** 10.3389/fphar.2018.00682

**Published:** 2018-06-26

**Authors:** Cuicui Yang, Xuelian Li, Wenbin Gao, Qi Wang, Li Zhang, Yali Li, Lin Li, Lan Zhang

**Affiliations:** ^1^Department of Pharmacy, Xuanwu Hospital of Capital Medical University, Beijing, China; ^2^Beijing Institute for Brain Disorders, Beijing, China; ^3^Beijing Engineering Research Center for Nerve System Drugs, Beijing, China; ^4^Key Laboratory for Neurodegenerative Diseases of Ministry of Education, Beijing, China; ^5^Institute of Clinical Pharmacology, Guangzhou University of Chinese Medicine, Guangzhou, China

**Keywords:** cornel iridoid glycoside, Alzheimer’s disease, tau phosphorylation, protein phosphatase 2A, glycogen synthase kinase-3β, protein phosphatase methylesterase-1

## Abstract

Neurofibrillary pathology contributes to neuronal dysfunction and correlates with the clinical progression of Alzheimer’s disease (AD). Tau phosphorylation is mainly regulated by a balance of glycogen synthase kinase-3β (GSK-3β) and protein phosphatase 2A (PP2A) activities. Cornel iridoid glycoside (CIG) is a main component extracted from *Cornus officinalis*. The purpose of this study was to investigate the effects of CIG on GSK-3β and PP2A, thus to explore the mechanisms of CIG to inhibit tau hyperphosphorylation. The rat model of tau hyperphosphorylation was established by intraventricular injection of wortmannin and GF-109203X (GFX) to activate GSK-3β. The results showed that intragastrical administration of CIG inhibited tau hyperphosphorylation in the brain of rats induced by wortmannin/GFX. The results *in vivo* and *in vitro* exhibited that CIG inhibited tau hyperphosphorylation and GSK-3β over-activation. In the mechanism of action, CIG’s attenuating GSK-3β activity was found to be dependent on PI3K/AKT signaling pathway. PP2A catalytic C subunit (PP2Ac) siRNA abrogated the effect of CIG on PI3K/AKT/GSK-3β. Additionally and crucially, we also found that CIG inhibited the demethylation of PP2Ac at Leu309 *in vivo* and *in vitro*. It enhanced PP2A activity, decreased tau hyperphosphorylation, and protected cell morphology in okadaic acid (OA)-induced cell model *in vitro*. PP2Ac siRNA abated the inhibitory effect of CIG on tau hyperphosphorylation. Moreover, CIG inhibited protein phosphatase methylesterase-1 (PME-1) and demethylation of PP2Ac, enhanced PP2A activity, and decreased tau hyperphosphorylation in PME-1-transfectd cells. Taken together, CIG inhibited GSK-3β activity via promoting P13K/AKT and PP2A signaling pathways. In addition, CIG also elevated PP2A activity via inhibiting PME-1-induced PP2Ac demethylation to inhibit GSK-3β activity, thus regulated the cross-talk between GSK-3β and PP2A signaling and consequently inhibited tau hyperphosphorylation. These results suggest that CIG may be a promising agent for AD therapy.

## Introduction

Alzheimer’s disease (AD) is the most common neurodegenerative disease in the elderly. The presence of neurofibrillary tangles (NFTs) in neurons is one of the main characteristic pathological events in AD. Data strongly suggest that neurofibrillary pathology contributes to neuronal dysfunction and correlates with the clinical progression of AD (Holtzman et al., 2011; Murray et al., 2015). NFTs are composed of abnormally hyperphosphorylated tau protein. Tau phosphorylation is regulated by several kinases and phosphatases (Lee et al., 2001; Hardy, 2006). Potentially novel strategies have aimed at targeting tau hyperphosphorylation in neurodegenerative disease, suggesting that kinase inhibitors or phosphatase activators will be a potential therapy target (Hanger et al., 2009; Clavaguera et al., 2014).

Glycogen synthase kinase-3β (GSK-3β) is a proline-directed serine/threonine protein kinase, and highly expressed in the central nervous system (CNS) (Woodgett, 1990). GSK-3 is one phosphorylation enzyme that enables intracellular tau phosphorylation, with excessive tau phosphorylation eliminating microtubule polymerization in the same way as with paired helical filament tau (PHF tau) (Liu et al., 2002; Takashima, 2012). Studies show that over-expression of GSK-3β in transgenic mice leads to tau hyperphosphorylation and memory deficits (Jope et al., 2007).

Protein phosphatase 2A (PP2A) is the major serine/threonine protein phosphatase, accounting for approximately 70% of the total tau phosphatase activity in human brain (Liu et al., 2005; Zimmer et al., 2016). PP2A activity is decreased in AD patients (Vogelsberg-Ragaglia et al., 2001). PP2A consists of a structural A subunit, a regulatory B subunit, and a catalytic C subunit (PP2Ac). The activity of PP2A is regulated by several post-translational modifications of PP2Ac, including phosphorylation and methylation (Sontag et al., 2004a). There is cross-talk between GSK-3β and PP2A signaling (Wang et al., 2015). GSK-3β regulates PP2Ac methylation via regulating protein phosphatase methylesterase-1 (PME-1) and leucine carboxyl methyltransferase-1 (LCMT-1); and PP2A dephosphorylates GSK-3β at Ser9 (Wang et al., 2015). There is no effective drug to activate PP2A or inhibit GSK-3β entering clinical trials (McGeer and McGeer, 2013).

*Cornus officinalis* Sieb. et Zucc is a member of the Cornaceac family. This traditional Chinese medicinal herb is included in treatments for dementia and other age-related diseases in China (Zhang et al., 2012; Jiang et al., 2013; Han et al., 2014). Cornel iridoid glycoside (CIG) is a main component extracted from *Cornus officinalis*. Previous studies in our laboratory indicated that CIG effectively improved memory ability and promoted neuronal survival through increasing the expression of synaptophysin and neurotrophic factors in cholinergic deficit AD-like model rats induced by fimbria-fornix transection (Zhao et al., 2010). Our resent study found that CIG treatment ameliorated the cognitive impairment and inhibited tau hyperphosphorylation in the hippocampus and striatum of senescence-accelerated mouse prone 8 (SAMP8) model (Ma et al., 2016). Our previous work also demonstrated that CIG attenuated tau hyperphosphorylation partially by enhancing PP2A activity in an AD-like cell model (Yang et al., 2013), and inhibited the demethylation of PP2A catalytic subunit C in the hippocampus of rat model induced by intracerebroventricular injection with OA (data not shown). It has been suggested that GSK-3β may play an important role in AD pathogenesis. In view of the important role of GSK-3β in tau hyperphosphorylation, in this study, we explored the effect of CIG on GSK-3β pathway and analyzed whether CIG exerts its effect via regulating cross-talk between GSK-3β and PP2A signaling.

## Materials and Methods

### Drugs

Cornel iridoid glycoside was extracted from *Cornus officinalis* in our laboratory as described previously (Yao et al., 2009; Zhao et al., 2010), and the purity of CIG was 70% determined by RP-HPLC assay, in which morroniside accounted for 67% and loganin 33%. Memantine was purchased from H. Lundbeck A/S, Denmark.

### Animals and Intraventrical Injection of Wortmannin/GFX

Male Wistar rats (weight 200–250 g) were purchased from Beijing Vital River Experimental Animal Co., Beijing, China. Rats were housed at a temperature between 20°C and 25°C with a 12/12-h dark/light cycle and in specific pathogen-free (SPF) conditions. For the entire duration of the experiment, the rats had free access to food and water. All animal care and experimental procedures were performed according to the requirements of the Provisions and General Recommendations of Chinese Experimental Animal Administration Legislation, and were approved by Bioethics Committee of Xuanwu Hospital of Capital Medical University.

Rats were anesthetized with chloral hydrate. After the scalp was incised, a 25 μl syringe (Hamilton, Switzerland) was stereotactically placed into the left ventricle at the co-ordinates for bregma and dura of AP-0.8, L-1.5 and V-4 (in mm). 200 μM wortmannin (from Enzo Life Sciences, United States) and 200 μM GF-109203X (GFX; from Sigma Chemical Co., United States) were dissolved in normal saline. A total volume of 10 μl of wortmannin/GFX in combination or normal saline (as sham control) was injected into the left cerebral ventricle of rats.

### Animal Grouping

Rats were randomly divided into 6 groups, *n* = 13 each group: sham control group, sham control + CIG (60 mg/kg) group, wortmannin/GFX model group, wortmannin/GFX + CIG (60 mg/kg) group, wortmannin/GFX + CIG (120 mg/kg) group, wortmannin/GFX + memantine (3 mg/kg). The doses of CIG was selected according to our previous studies (Yao et al., 2009; Zhao et al., 2010). Memantine was taken as the positive control drug because there were some reports indicating that memantine (a NMDA receptor antagonist) could also inhibit tau hyperphosphorylation (De Sarno et al., 2006).

**FIGURE 1:**
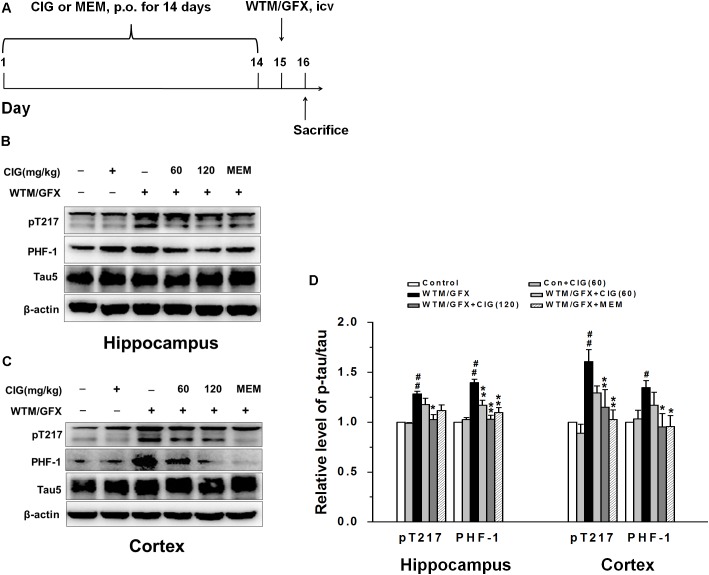
Cornel iridoid glycoside (CIG) inhibits tau hyperphosphorylation and GSK-3β induced by wortmannin/GFX in rats. **(A)** Flow chart of experimental schedule. **(B–D)** The phosphorylated tau protein at Thr217, PHF-1 and total tau5 in the hippocampus and cortex detected by western blotting assay. β-actin was used as an internal control, and the expression level of control group was set as 100%. Data are expressed as mean ± SD; *n* = 4 per group. *^#^P* < 0.05, *^##^P* < 0.01, the model group compared with the control group; *^∗^P* < 0.05, ^∗^*^∗^P* < 0.01, drug groups compared with the model group. WTM, wortmannin; GFX, GF-109203X; MEM, memantine, 3 mg/kg; p.o., intragastric administration; icv, intracerebroventricular injection.

**Figure 1A** shows the flow chart of experimental schedule. Rats were administered intragastrically with CIG or vehicle (distilled water) for 14 days. On the 15th day, wortmannin/GFX was intraventrically injected to the rats; sham control group and sham control + CIG group were injected with normal saline. Twenty-four hours after injection, the rats were then anesthetized and sacrificed, and the brains were taken out for further studies.

### Cell Culture

Human neuroblastoma SK-N-SH cells were cultured in RPMI-1640 supplemented with 10% (v/v) fetal bovine serum (FBS; from Gibco Invitrogen, Carlsbad, CA, United States). Mouse neuroblastoma N2a cells were purchased from National Infrastructure of Cell Line Resource (Beijing, China), and cultured in 45% Dulbecco’s modified Eagle’s medium (DMEM; from Gibco Invitrogen) and 45% OPTI-MEM supplemented with 10% FBS. Human embryonic kidney cells (HEK) 293T were obtained from Dr. Yu Zhang (Xuanwu Hospital, Beijing, China). HEK-293T cells were cultured in 90% DMEM supplemented with 10% FBS. All cells were kept in a humidified atmosphere of 5% CO_2_ and 95% air at 37°C.

### Transfection of GSK-3β Plasmid and Small Interfering RNA (siRNA)

Plasmid expressing hemagglutinin (HA)-tagged wild type GSK-3β was obtained from Jiangsu Key Laboratory of Neuroregeneration, Nantong, China. HEK-293T cells were plated to be at 60–70% confluence overnight, and transiently transfected with wtGSK-3β plasmid (final concentration 1.2 μg/ml) and human tau40 plasmid (1 μg/ml) using Lipofectamine 2000 (Invitrogen, Carlsbad, CA, United States) according to the manufacturer’s instructions. After incubation at 37°C for 24 h, the medium was replaced and the cells were incubated with CIG for 24 h after transfection.

HEK-293T cells were transfected with human tau40 plasmid together with human PP2Ac siRNA (QIAGEN, Hilden, Germany). The detailed protocol followed Attractene Transfection Reagent Handbook (QIAGEN). In brief, 2.5 × 10^5^ HEK-293T cells per well were seeded in a 12-well plate in 1 ml of an appropriate culture medium containing serum and antibiotics. Then each well was resuspended in 1 ml siRNA transfection medium with 3 μl transfection reagent, 0.8 μg human tau40 plasmid, and 20 nM PP2A siRNA; the control group was transfected with 20 nM non-silencing control siRNA. Cells were rinsed twice with cold PBS (pH 7.4) 24 h after siRNA transfection. Different concentrations of CIG were incubated with the cells for 24 h, then the cells were harvested.

### Lentivirus Vector Transduction

Transduction of N2a cells was carried out with indicated cell numbers and infection of the vector in the presence of polybrene. Using this system, high titer lentivirus (Shanghai Genechem Co., Shanghai, China) at titers of up to 2 × 10^8^ transducing units/ml (TU/ml) was successfully generated. Twenty-four hours later, cells were rinsed twice with cold PBS (pH 7.4), and CIG at different concentrations was incubated with cells for 24 h.

### Western Blotting Assay

For immunoblotting analysis, cells or tissues were sonicated in RIPA buffer (Beyotime Co., Jiangsu, China). The resulting lysates were mixed with 5× loading buffer and boiled for 5 min. Lysates were centrifuged at 12,000 *g* for 30 min at 4°C, and the supernatants were collected for analysis. The protein concentration was detected using an RC-DC protein assay according to manufacturer’s instructions (Bio-Rad, United States). Proteins were separated on 10% sodium dodecyl sulfate-polyacrylamide (SDS-PAGE) gels at 120 V for 1.5 h and transferred to nitrocellulose membrane. Membranes were blocked with 5% dry milk in Tris-buffered saline with 0.05% Tween-20 (TBST) and probed with primary antibodies that were incubated overnight at 4°C (Yang et al., 2013). The primary antibodies used are listed in **Table 1**. Following this, blots were washed three times in TBST and incubated with the appropriate secondary antibodies for 2 h. After washing, the blots were developed using the enhanced chemiluminescence method (ECL; Pierce, Rockford, IL, United States) and protein bands were visualized by the chemiluminescent detection system. Densitometric quantification of the protein bands were analyzed using TINA software (Raytest Isotopenme Bgerate GmbH, Straubenhardt, Germany).

**Table 1:** Primary antibodies used in this study.

Antibody	Action site	Source
pS199/202	Ser199/ Ser202	Invitrogen
pT212	Thr212	Invitrogen
pT205	Thr205	Invitrogen
pT217	Thr217	Invitrogen
pS396	Ser396	Invitrogen
PHF-1	Ser396/Ser404	Abcam
Tau-5		Abcam
p-GSK-3β	Ser9	Cell signaling
GSK-3β		Santa cruz
p-AKT	Ser473	Cell signaling
AKT		Cell signaling
Demethylated PP2Ac	Leu307	Millipore
Methylated PP2Ac	Leu307	Millipore
PME-1		Santa cruz
LCMT-1		Abcam
p-PP2A-Cα/β	Tyr307	Santa cruz
PP2A		Santa cruz
PI3K p110α		Cell signaling
PI3K p85 p-PI3K p85	Tyr607	Cell signaling Cell signaling
β-actin		ZSGB-BIO
GAPDH		ZSGB-BIO

### Measurement of PP2A Activity

PP2A activity in the cell extracts was detected using V2460 kit (Promega, Madison, WI, United States) according to the manufacturer’s protocol (Sun et al., 2012). Briefly, cells were rinsed twice with cold PBS (pH 7.4) and lysed in a cooled buffer for 20 min. Lysates were centrifuged at 12,000 *g* at 4°C for 1 h, and the supernatants were collected for the measurement of PP2A activity. Endogenous free phosphates were removed and the protein content of the extracts was normalized across all samples. Enzyme samples were incubated with a chemically synthesized phosphopeptide in a 96 well plate for 30 min at 30°C. The reaction was stopped by adding 50 μl molybdate dye/additive mixture. Phosphate release from the substrate was detected by measuring the absorbance of the molybdate-malachite green-phosphate complex at 630 nm. PP2A activity was evaluated as the release of phosphate per μ⋅protein and per minute (pmol/μg/min).

### Statistical Analysis

All data were expressed as mean ± standard deviation (SD) and analyzed using the software package SPSS 16.0 (SPSS, Inc., Chicago, IL, United States). Results were assessed using one-way analysis of variance (ANOVA) followed by Tukey’s *post hoc* test. A probability of *P* < 0.05 was considered statistically significant.

## Results

### CIG Attenuates Tau Hyperphosphorylation Induced by Wortmannin/GFX in Rats

In the present study, we used wortmannin (an inhibitor of PI3K) together with GF-109203X (GFX, an inhibitor of protein kinase C) to establish tau hyperphosphorylation rat model of AD. **Figure 1A** shows the flow chart of experimental schedule. We measured the level of tau phosphorylation. Western blotting analysis exhibited that the expression of phosphorylated tau at Thr205 (pT205) and Ser396/404 (PHF-1) was increased in the hippocampus and cortex of wortmannin/GFX model rats (*P* < 0.05, *P* < 0.01), while CIG (120 mg/kg) decreased the hyperphosphorylated tau at these sites induced by wortmannin/GFX (*P* < 0.05, *P* < 0.01; **Figures 1B–D**). Memantine (MEM) at the dose of 3 mg/kg exhibited similar effects to CIG.

### CIG Inhibits GSK-3β Activity Depending on Activation of PI3K/AKT Signaling Pathway

**FIGURE 2:**
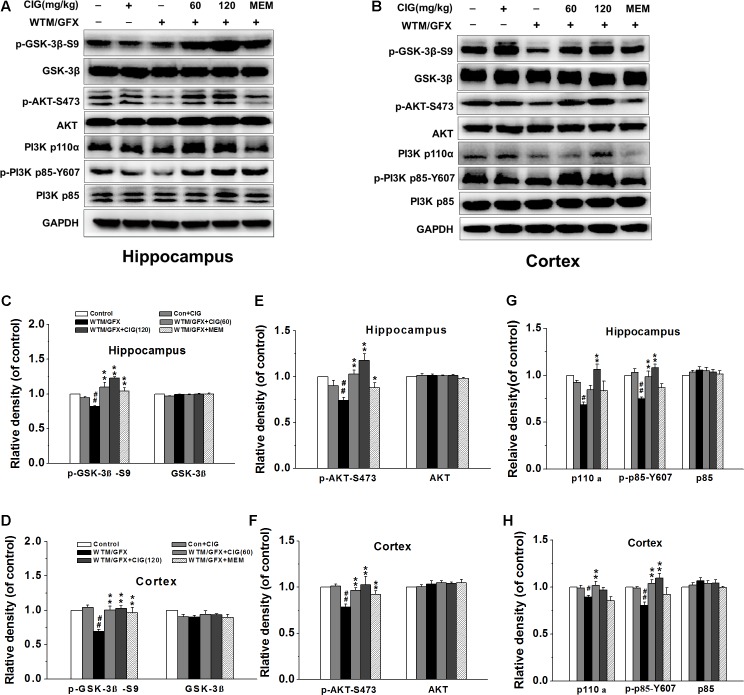
Effects of CIG on GSK-3β and PI3K/Akt signaling pathway in the hippocampus and cortex in wortmannin/GFX model rats. **(A,B)** The representative images of GSK-3β, Akt and PI3K detected by western blotting analysis. **(C,D)** Quantitative analysis of GSK-3β and phosphorylated GSK-3β at Ser9. **(E,F)** Quantitative analysis of AKT and phosphorylated AKT at Ser473. **(G,H)** Quantitative analysis of PI3K p110α, p85 and phosphorylated p85 at Tyr607. β-actin was used as an internal control, and the expression level of control group was set as 100%. Data are expressed as mean ± SD.; *n* = 4 per group. *^#^P* < 0.05, *^##^P* < 0.01, the model group compared with the control group; *^∗^P* < 0.05, ^∗^*^∗^P* < 0.01, the drug groups compared with the model group. WTM, wortmannin; GFX, GF-109203X; MEM, memantine, 3 mg/kg.

To explore the mechanism of CIG on the overactivation of GSK-3β, we measured the related protein in PI3K/AKT signaling pathway. **Figures 2A,B** display the representative images of GSK-3β, PI3K and AKT expression in the hippocampus and cortex of rats detected by western blotting assay. The level of phosphorylated GSK-3β at Ser9 (representing the inactivated form of GSK-3β) was significantly declined in wortmannin/GFX model group (*P* < 0.01), while the treatment with CIG at the doses of 60 and 120 mg/kg obviously increased the level of the Ser9-phosphorylated GSK-3β in the hippocampus and cortex of wortmannin/GFX model rats (*P* < 0.01; **Figures 2C,D**), suggesting that CIG may reduce the activity of GSK-3β.

It has been known that the phosphorylation of GSK-3β at Ser9 is increased by the activation of phosphatidylinositol 3-kinase (PI3K) and serine/threonine kinase (AKT) (Kitagishi et al., 2014). In the present study, the results displayed that wortmannin/GFX induced a remarkable decrease in phosphorylated-Akt at Ser473 in the hippocampus and cortex of rats (*P* < 0.01), whereas CIG significantly elevated the level of phosphorylated-Akt at Ser473 in wortmannin/GFX rats (*P* < 0.01; **Figures 2E,F**).

To elucidate whether Akt phosphorylation was due to activation of PI3K, we further analyzed the levels of PI3K’s catalytic subunitp110α and regulatory subunit p85. The results showed that wortmannin/GFX markedly declined the levels of PI3K p110α and phosphorylated p85 at Tyr607 in the hippocampus and cortex of rats (*P* < 0.05, *P* < 0.01); the treatment with CIG obviously increased the levels of PI3K p110α and phosphorylated p85 at Tyr607 in rats exposed to wortmannin/GFX (*P* < 0.05, *P* < 0.01; **Figures 2G,H**).

### CIG Inhibits GSK-3β Through Decreasing PP2Ac Demethylation to Attenuate Tau Hyperphosphorylation

**FIGURE 3:**
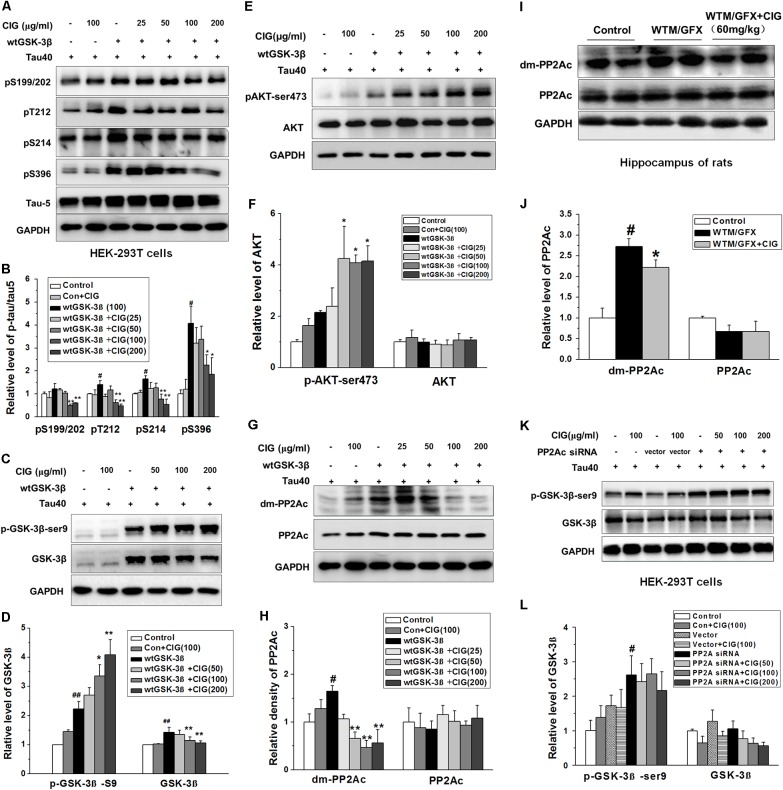
Cornel iridoid glycoside inhibits GSK-3β through decreasing PP2Ac demethylation to suppress tau hyperphosphorylation. **(A,B)** The phosphorylation at different sits of tau protein in HEK-293T cells transfected with wild type GSK-3β plasmid together with human tau40 plasmid was detected by western blotting assay. CIG at different doses was incubated with transfected cells for 24 h. **(C,D)** The levels of GSK-3β and phosphorylated GSK-3β at Ser9 site in GSK-3β-transfected cells. **(E,F)** Quantitative analysis of AKT and phosphorylated AKT at Ser473 in the cells transfected with GSK-3β plasmid. **(G,H)** The levels of PP2A catalytic C subunit (PP2Ac) and demethylated PP2Ac (dm-PP2Ac) at Leu309 site in cells transfected with GSK-3β plasmid. **(I,J)** The levels of PP2Ac and dm-PP2Ac at Leu309 in the hippocampus of rats intraventricularly injected with wortmannin/GFX. **(K,L)** The levels of GSK-3β and phosphorylated GSK-3β at Ser9 in HEK-293T cells transfected with PP2Ac siRNA. GAPDH was used as an internal control, and the expression level of control group was set as 100%. We detected p-tau and PP2Ac in the same electrophoresis and on the same membrane using different antibodies, and used the same loading control GAPDH in **(A,G)**. Data are present as mean ± SD; *n* = 3–4 each group. *^#^P* < 0.05, *^##^P* < 0.01, the model group compared with the control group; *^∗^P* < 0.05, *^∗∗^P* < 0.01, drug groups compared with the model group. WTM, wortmannin; GFX, GF-109203X; MEM, memantine, 3 mg/kg.

In order to further explore the mechanism of CIG’s inhibiting GSK-3β over-activation to attenuate tau hyperphosphorylation, we transfected wild type GSK-3β plasmid together with human tau40 plasmid into HEK-293T cells. **Figures 3A,B** show that the expression of phosphorylated tau at multiple sites was obviously increased in GSK-3β-transfected cells (*P* < 0.05); CIG (100 and 200 μg/ml) treatment for 24 h significantly reduced the levels of tau phosphorylation at Ser199/202, Thr212, Ser214, and Ser396 (*P* < 0.05, *P* < 0.01). As shown in **Figures 3C,D**, total GSK-3β was markedly up-regulated in cells transfected with GSK-3β (*P* < 0.01); the treatment with CIG significantly inhibited the over-expression of GSK-3β and increased the phosphorylation of GSK-3β at Ser9 compared with the transfected cells without treatment (*P* < 0.05, *P* < 0.01). In addition, the level of phosphorylated-Akt at Ser473, which is an upstream kinase for phosphorylating GSK-3β, was significantly increased by CIG treatment (*P* < 0.05; **Figures 3E,F**).

Interestingly, we found that GSK-3β transfection also induced a remarkable elevation in demethylation of PP2A catalytic C subunit (dm-PP2Ac) (*P* < 0.05); the demethylation of PP2Ac was significantly decreased by CIG treatment in cells transfected with GSK-3β plasmid (*P* < 0.01; **Figures 3G,H**). The results *in vivo* also showed that intraventrical injection of wortmannin/GFX obviously increased the demethylation of PP2Ac in the hippocampus of rats (*P* < 0.05); intragastric administration of CIG significantly decreased the demethylation of PP2Ac induced by wortmannin/GFX in rats (*P* < 0.05; **Figures 3I,J**).

In order to confirm whether the effects of CIG on GSK-3β were mediated by PP2Ac, we transferred human PP2Ac siRNA into HEK-293T cells. The results showed that the phosphorylation of GSK-3β at Ser9 was increased in PP2Ac siRNA-transfected cells (*P <* 0.05; **Figures 3K,L**), which was similar to that in GSK-3β-transfected cells (**Figures 3C,D**). However, CIG had no effect on GSK-3β phosphorylation at Ser9 after PP2Ac was knocked down (**Figures 3K,L**). These results suggest that CIG may inhibit GSK-3β activity via decreasing PP2Ac demethylation.

### CIG Enhances PP2A Activity via Inhibiting PP2Ac Demethylation Directly

**FIGURE 4:**
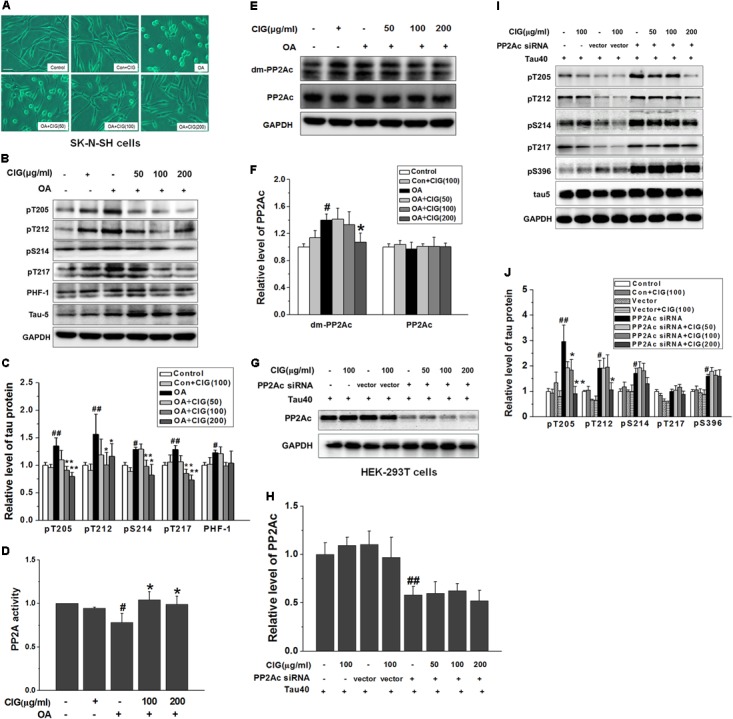
Cornel iridoid glycoside enhances PP2A activity through inhibiting PP2Ac demethylation directly. **(A)** The morphological changes of human neuroblastoma SK-N-SH cells exposed to okadaic acid (OA) at the dose of 20 nM for 6 h were observed under contrast microscope (Scale bar = 50 μm). CIG at different doses was preincubated with cells for 24 h. **(B,C)** The phosphorylation of different sites of tau protein in OA-induced model cells was detected by western blotting assay. **(D)** The activity of PP2A was measured by a biochemical assay (*n* = 6 each group). **(E,F)** The levels of PP2Ac and demethylated PP2Ac (dm-PP2Ac) at Leu309 site were detected by western blotting. **(G,H)** The level of PP2Ac in HEK-293 cells transfected with PP2Ac siRNA. CIG was incubated with transfected cells for 24 h. **(I,J)** The phosphorylation of tau protein in PP2Ac siRNAc-transfected cells. GAPDH was used as an internal control, and the expression level of control group was set as 100%. We detected p-tau and PP2Ac in the same electrophoresis and on the same membrane using different antibodies, and used the same loading control GAPDH in **(B,E)**. Data represent mean ± SD; *n* = 3–4 each group. *#P* < 0.05, *##P* < 0.01, the model group compared with the control group; ^∗^*P* < 0.05, ^∗∗^*P* < 0.01, drug groups compared with the model group.

In order to investigate whether CIG had effect on PP2A, we used OA to inhibit PP2A activity. **Figure 4A** shows that normal human neuroblastoma SK-N-SH cells spread well, and incubation of CIG did not influence the morphology of the normal cells. After exposure to OA for 6 h, the cell bodies became round and the axons were shortened. Preincubation of CIG (100 and 200 μg/ml) for 24 h with cells prevented the damage induced by OA and improved the morphology of cells.

As shown in **Figures 4B,C**, tau phosphorylation at multiple sites was significantly increased in OA-treated SK-N-SH cells (*P* < 0.05, *P* < 0.01). Pretreatment with CIG (100 and 200 μg/ml) markedly decreased the phosphorylated tau at sites Thr205, Thr212, Ser214 and Thr217 in the cells exposed to OA (*P* < 0.05, *P* < 0.01), and had a trend of reducing PHF-1 (Ser396/404).

As an inhibitor of PP2A, OA induced a decrease in PP2A activity (*P* < 0.05) and an increase in demethylation of PP2Ac at Leu309 in SK-N-SH cells (*P* < 0.05); CIG significantly elevated PP2A activity (*P* < 0.05; **Figure 4D**) and reduced demethylation of PP2Ac at Leu309 in OA-exposed cells (*P* < 0.05; **Figures 4E,F**). The results suggest that CIG may increase PP2A activity through inhibiting demethylation of PP2Ac.

Then we transfected PP2Ac siRNA into HEK293T cells, and found that PP2Ac expression was obviously decreased and tau phosphorylation at multiple sites was markedly increased in siRNA-transfected cells (*P* < 0.05, *P* < 0.01); CIG (100 and 200 μg/ml) did not reverse the decline of PP2Ac expression (**Figures 4G,H**) and the elevation of tau phosphorylation at Ser214, Thr217 and Ser396, and partially reversed tau hyperphosphorylation at Thr205 and Thr212 in PP2Ac siRNA-transfected cells (**Figures 4I,J**). These findings mean that the ability of CIG to activate PP2A and to inhibit tau phosphorylation was nearly abolished after PP2Ac was knocked down. Results demonstrated that CIG increased PP2A activity via decreasing demethylation of PP2Ac.

### CIG Inhibits Demethylation of PP2Ac via Decreasing PME-1

**FIGURE 5:**
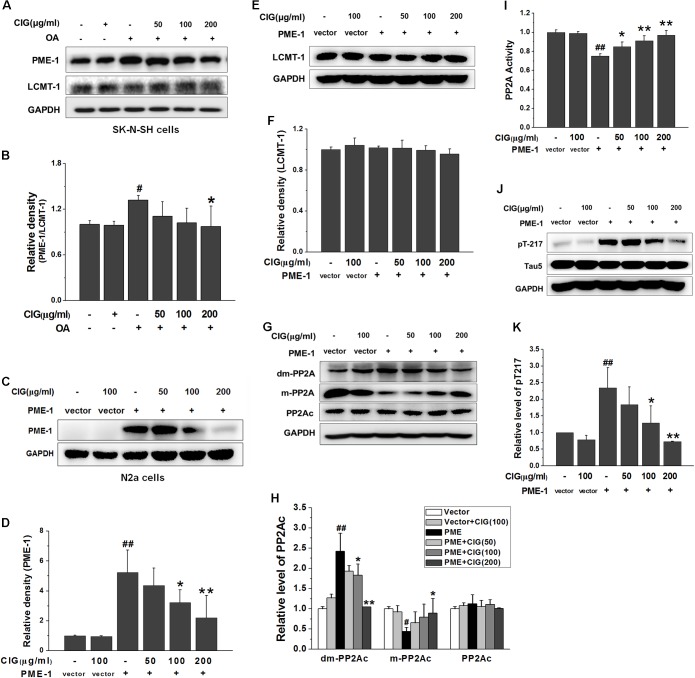
Cornel iridoid glycoside inhibits demethylation of PP2Ac via decreasing PME-1. **(A,B)** The expression of PME-1 and LCMT-1 in OA-treated SK-N-SH cells, and the ratio of PME-1/LCMT-1. **(C,D)** The expression of PME-1 in mouse neuroblastoma N2a cells transduced with PME-1 genetic materials delivered by recombined lentivirus vector. CIG was incubated with the transduced cells for 24 h. **(E,F)** The expression of LCMT-1 in N2a cells transduced with PME-1 genetic materials. **(G,H)** The expression of demethylated PP2Ac (dm-PP2Ac) and methylated PP2Ac (m-PP2Ac). **(I)** The activity of PP2A measured by a biochemical assay, *n* = 6 per group. **(J,K)** The phosphorylation of tau at Thr217 (pT217). GAPDH was used as an internal control, and the expression level of control group was set as 100%. Data are expressed as mean ± SD; *n* = 3–4 each group. *^#^P* < 0.05, *^##^P* < 0.01, the model group compared with the control group; ^∗^*P* < 0.05, ^∗∗^*P* < 0.01, drug groups compared with the model group.

It has been reported that PP2A activity is increased by methylation of PP2Ac. The methylation of PP2Ac is regulated by protein phosphatase methylesterase-1 (PME-1) which promotes demethylation of PP2A, and leucinecarboxyl methyltransferase-1 (LCMT-1) which catalyzes methylation of PP2A (Stanevich et al., 2011; Kaur and Westermarck, 2016). In the present study, OA exposure markedly increased the ratio of PME/LCMT in SK-N-SH cells (*P* < 0.05); CIG treatment significantly decreased the ratio of PME/LCMT in OA-exposed cells (*P* < 0.05; **Figures 5A,B**).

To further investigate whether CIG’s inhibiting demethylation of PP2Ac was mediated by PME-1, we used recombined lentivirus vector to deliver PME-1 genetic materials into mouse neuroblastoma N2a cells. The results showed that PME-1 was expressed at high level in N2a cells after transduction (*P* < 0.01). Incubation of CIG (100 and 200 μg/ml) with the transduced cells for 24 h significantly reduced PME-1 expression (*P* < 0.05, *P* < 0.01; **Figures 5C,D**). There was no obvious difference in LCMT-1 expression among all groups in PME-1-transduced cells (**Figures 5E,F**). The demethylation of PP2Ac was increased and methylation of PP2Ac was decreased after PME-1 transduction (*P* < 0.05, *P* < 0.01); CIG treatment elevated methylation of PP2Ac (the active form) and suppressed demethylation of PP2Ac (the inactive form) in transduced cells (*P* < 0.05, *P* < 0.01; **Figures 5G,H**). PME-1 transduction also induced a decrease in PP2A activity (*P* < 0.01) and an increase in tau hyperphosphorylation at Thr217 (*P* < 0.01); CIG treatment elevated PP2A activity (*P* < 0.05, *P* < 0.01; **Figure 5I**) and thus inhibited tau hyperphosphorylation at Thr217 in PME-1-transduced N2a cells (*P* < 0.05, *P* < 0.01; **Figures 5J,K**). These results indicated that CIG inhibited demethylation of PP2Ac via decreasing PME-1, thus elevated PP2A activity and reduced tau hyperphosphorylation.

### CIG Inhibits Demethylation of PP2Ac Not Entirely Dependent on LCMT-1

**FIGURE 6:**
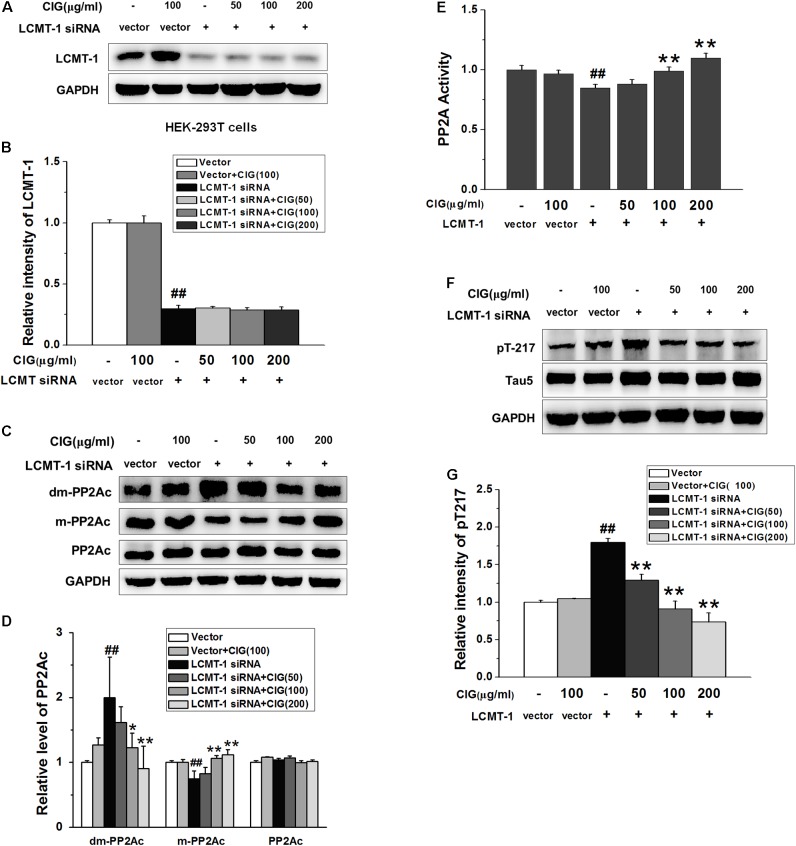
Cornel iridoid glycoside inhibits demethylation of PP2Ac not entirely dependent on LCMT-1. **(A,B)** The expression of LCMT-1 in LCMT-1 siRNA-transfected HEK-293T cells detected by western blotting assay. CIG was incubated with the transfected cells for 24 h. **(C,D)** The expression of demethylated PP2Ac (dm-PP2Ac) and methylated PP2Ac (m-PP2Ac) in the transfected cells. **(E)** The activity of PP2A measured by a biochemical assay, *n* = 6 each group. **(F,G)** The phosphorylation of tau at Thr217 (pT217). GAPDH was used as an internal control, and the expression level of control group was set as 100%. Data represent mean ± SD; *n* = 3–4 each group. *^#^P* < 0.05, *^##^P* < 0.01, the model group compared with the control group; ^∗^*P* < 0.05, ^∗∗^*P* < 0.01, drug groups compared with the model cells.

In order to observe whether CIG’s inhibiting demethylation of PP2Ac was regulated by LCMT-1, we transfected LCMT-1 siRNA into HEK-293T cells. The results exhibited that LCMT-1 expression was significantly decreased in LCMT-1 siRNA-transfected cells (*P* < 0.01); CIG (100 and 200 μg/ml) did not reverse the decline of LCMT-1 expression (**Figures 6A,B**). As shown in **Figures 6C,D**, the demethylation of PP2Ac was increased and the methylation of PP2Ac was decreased in LCMT-1 siRNA-transfected cells (*P* < 0.01); CIG reversed the high demethylation and low methylation of PP2Ac (*P* < 0.05, *P* < 0.01) although LCMT-1 had been knocked down. The transfection of LCMT-1 siRNA also decreased the activity of PP2A (*P* < 0.01) and increased tau hyperphosphorylation at Thr217 (*P* < 0.01); CIG elevated PP2A activity (*P* < 0.01; **Figure 6E**) and inhibited tau hyperphosphorylation at Thr217 in LCMT-1 siRNA-transfected cells (*P* < 0.01; **Figures 6F,G**). These results suggest that CIG’s inhibiting demethylation of PP2Ac was not entirely dependent on LCMT-1.

## Discussion

In this study, we addressed the question of whether CIG could inhibit tau hyperphosphorylation via regulating the crosstalk between GSK-3β and PP2A signaling pathway. Memory deterioration and synapse damage with accumulation of hyperphosphorylated tau are hallmark lesions of AD (Li et al., 2012). Under pathogenic conditions, hyperphosphorylation occurs at many sites of tau, promoting microtubule dissociation and impairment of axonal transport and synaptic function, which collectively drive neuronal toxicity and cell death (Kolarova et al., 2012; Pascual et al., 2017). GSK-3β is implicated in multiple cellular processes and has been linked to the pathogenesis of AD (Li et al., 2006). The increased level of GSK-3β has been found in post-mortem analysis of brains from AD patients and is correlated with the progression of NFT and neurodegeneration (Pei et al., 1997; Leroy et al., 2002). Inhibition of GSK-3β leads to neuroprotective effects and a reduction in tau hyperphosphorylation (Yu and Lin, 2016). Activated phosphoinositol-3 kinase (PI3K) induces the activation of AKT, which phosphorylates various biological substrates, including GSK-3β. Wortmannin (a specific inhibitor of PI3K110a) and GF-109203X (GFX, a specific inhibitor of protein kinase C) can activate GSK-3β via inhibiting phosphorylation of GSK-3β at Ser9 site (the inactive form) (Wymann et al., 1996). Therefore, we used wortmannin/GFX to establish an AD-like animal model through activating GSK-3β in the present study. The results showed that intraventricular injection of wortmannin/GFX induced an increase in the expression of GSK-3β and a decrease in phosphorylated GSK-3β at Ser9 (may elevate the activity of GSK-3β), thus led to an increase in tau hyperphosphorylation in the hippocampus and memory impairment in rats. These results are consistent with other investors’ reports (Qian et al., 2010; Wang et al., 2015). Our experiment also demonstrated that intragastric administration of CIG prevented above pathological changes in the brain.

It has been reported that GSK-3β is the downstream target of PI3K/AKT signaling pathway. PI3K is a heterodimer composed of regulatory subunit p85 and catalytic subunit p110. The binding of p85 to phosphotyrosine residues of activated receptors is critical for the active conformation of p110 (Vivanco and Sawyers, 2002). The conformation change in the heterodimer promotes PI3K activity, which results in synthesis of phosphatidylinositol trisphosphate (PIP3) and consequently enhances AKT activity (Jeong et al., 2017). Activated AKT inhibits the downstream substrate GSK-3β, thus suppresses tau hyperphosphorylation. PI3K/AKT signaling pathway dysfunction causes GSK-3β activity increase and leads to tau hyperphosphorylation (Kitagishi et al., 2014). In the present study, CIG increased the expression of PI3K p110 and the phosphorylation of PI3K p85 at Tyr607, and elevated the phosphorylation of AKT at Ser473, thus decreased the activity of GSK-3β represented by increasing the phosphorylation of GSK-3β at Ser9 in the hippocampus and cortex of wortmannin/GFX rats and in GSK-3β-transfected cells. Results implied that CIG inhibited GSK-3β via activating PI3K/AKT signaling pathway, and this may be one of mechanisms by which CIG inhibits tau hyperphosphorylation. In addition, the results from GSK-3β-transfected cells showed that CIG inhibited tau hyperphosphorylation at Ser199/202, Thr212, Ser214, and Ser396, in which Ser199 and Ser396 have been reported as the most favorable sites of GSK-3β (Qian et al., 2010), while Thr212 and Ser214 are the most favorable sites of PP2A (2005; Qian et al., 2010). This reminded us that, besides inhibiting GSK-3β, CIG might also affect PP2A.

PP2A activity is decreased in AD brain compared with non-demented controls in frontal or temporal cortex (Gong et al., 1993, 1995). Decreased mRNA and protein expression of PP2A leads to the hyperphosphorylation of tau and the formation of NFTs, as well as neuron degeneration in AD (Vogelsberg-Ragaglia et al., 2001). The heterodimeric PP2A core enzyme consists of a 36 kDa catalytic subunit (or C subunit) and a 65 kDa scaffold subunit (or A subunit) (Voronkov et al., 2011). PP2A activity is regulated by post-translational modification mainly via methylation of its catalytic C subunit (PP2Ac). Increased demethylation of PP2Ac has been proposed to have a relationship with elevated level of tau hyperphosphorylation observed in AD (Zhang et al., 2008). Previous studies in our laboratory showed wortmannin/GFX led to an elevation in both GSK-3β and demethylation of PP2Ac. In the present study indicated that transfection of GSK-3β plasmid into HEK-293T cells induced an increase in demethylation of PP2Ac. These results are consistent with other investigators’ reports. Wang et al. have proposed that the cross talk between PI3K-AKT-GSK-3β and PP2A pathways determines tau hyperphosphorylation (Wang et al., 2015). Martin and Yao have reported that GSK-3β can inhibit PP2A activity via increasing demethylation of PP2Ac at Leu309 (Martin et al., 2011; Yao et al., 2012). In the present study, we found that CIG inhibited PP2Ac demethylation at Leu309 in GSK-3β-transfected cells, suggesting that CIG may inhibit tau hyperphosphorylation via regulating the balance between GSK-3β and PP2A.

We had investigated CIG enhanced PP2A activity independent of regulating GSK-3β (Yang et al., 2013). It has been reported that PP2A also regulates GSK-3β phosphorylation (Wang et al., 2015). PP2A regulates tau phosphorylation directly and also indirectly via activating GSK-3β (Qian et al., 2010). So we also examined whether CIG affected GSK-3β activity when PP2A was inhibited by PP2Ac siRNA. In the present study, we transfected PP2Ac siRNA into cells, and found that knockdown of PP2A induced an enhancement in GSK-3β phosphorylation at Ser9 thus inhibited GSK-3β activity, which is in consistent with other reports (Qian et al., 2010; Wang et al., 2015) CIG had no effect on the increase of GSK-3β phosphorylation in PP2Ac siRNA-transfected cells. These results suggest that CIG may affect GSK-3β activity by regulating PP2Ac demethylation.

In order to further determine whether CIG could regulate PP2A activity via inhibiting PP2Ac demethylation, we used OA to establish a cell model. OA is a selective inhibitor of PP2A and can induce tau hyperphosphorylation and accumulation (Gong et al., 2003; Ikema et al., 2015). Tau protein acts as structural stabilizers for the main nerve cell frame. Under disease conditions, tau protein induces disruption of axonal integrity (Hoover et al., 2010). In the present study, the results exhibited that OA induced a decrease in PP2A activity, thus leading to tau hyperphosphorylation and retraction of cell processes. These changes in OA model group are consistent with other investigators’ reports (Garcia et al., 2002; Tian et al., 2004; Ranjan and Heintz, 2006). CIG elevated PP2A activity, inhibited tau hyperphosphorylation and protected nerve cells from damage induced by OA. It has been reported that PP2A activity is regulated by post-translational modification mainly via methylation of PP2Ac (Eichhorn et al., 2009; Qian et al., 2010), and demethylation of PP2Ac at the Leu309 decreases PP2A activity (Sontag et al., 2004b). Our present study found that OA caused an increase in demethylation of PP2Ac, whereas CIG treatment prevented the elevation of PP2Ac demethylation induced by OA. After PP2Ac was knocked down by siRNA transfection, CIG could not attenuate tau hyperphosphorylation at several sites, which implied that PP2Ac siRNA abolished the effect of CIG on inhibiting tau hyperphosphorylation. These results indicated that CIG inhibited tau hyperphosphorylation mainly through increasing PP2A activity caused by decreasing demethylation of PP2Ac.

Carboxyl methylation of PP2Ac is regulated by the opposing activities of PME-1 (promoting demethylation of PP2A) and LCMT-1 (catalyzing methylation of PP2A) (Kalhor et al., 2001; Leulliot et al., 2004; Hwang et al., 2016). This process regulates the recruitment of specific regulatory B subunit to the (AC) core enzyme, thereby contributing to regulation of PP2A biogenesis and substrate specificity (Sontag et al., 2008). PME-1 directly binds to the active site of PP2A (Longin et al., 2004) and catalyzes removal of the methyl group, thus reversing the activity of LCMT-1 (Xing et al., 2008). In the present study, we transfected PME-1 genetic materials into nerve cells, and found that over-expression of PME-1 induced an increase in demethylation and a decrease in methylation of PP2Ac. CIG markedly inhibited PME-1 over-expression, declined demethylation of PP2Ac, elevated PP2A activity, thus inhibited tau hyperphosphorylation in PME-1-transfected cells.

It has been reported that LCMT-1 catalyzes methylation of PP2A, and loss of the methyltransferase gene causes phenotypes similar to those associated with over-expression of PP2A methylesterase (Wu et al., 2000). In our experiment, the transfection of LCMT-1 siRNA caused a decrease in methylation and an increase in demethylation of PP2Ac. However, knockdown of LCMT-1 by siRNA did not abolish the effects of CIG’s reducing demethylation and elevating methylation of PP2Ac. Similarly, CIG still enhanced PP2A activity and inhibited tau hyperphosphorylation in LCMT-1 siRNA-transfecte cells. These results suggest that CIG may regulate methylation of PP2Ac not entirely dependent on LCMT-1.

In our another study, we investigated the effects of CIG on amyloid and tau phosphorylation in APP/PS1/Tau transgenic (3xTg) mice. The results showed that CIG ameliorated learning and memory deficits, and decreased both Aβ deposition and tau hyperphosphorylation in the cerebral cortex and hippocampus of 3xTg mice (data not shown), suggesting that CIG could also act on Aβ mechanism besides tau phosphorylation.

In the present study, the purity of CIG was 70% by HPLC, and remaining 30% contained some small peaks, which were unable to be separated, purified and identified. It is possible that not all observed effects are due to glycoside. The effects of components in the remaining 30% on AD remain to be further investigated.

## Conclusion

**FIGURE 7:**
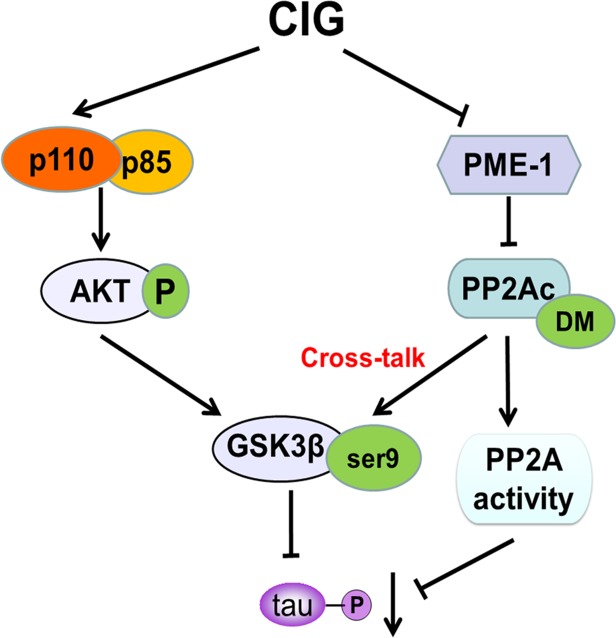
A proposed scheme shows the mechanisms of CIG to inhibit tau hyperphosphorylation.

The findings presented in this study provided novel mechanistic insights into how CIG inhibited tau hyperphosphorylation. Herein, we demonstrated for the first time that CIG inhibited GSK-3β activity through promoting PI3K/AKT signaling pathway. In addition, CIG could also elevated PP2A activity via inhibiting PME-1-induced PP2Ac demethylation to inhibit GSK-3β activity, thus regulated the cross-talk between GSK-3β and PP2A signaling and consequently inhibited tau hyperphosphorylation (**Figure 7**). These results suggest that CIG may be used as a promising drug for preventing the progression of AD.

## Author Contributions

LaZ and LL designed the experiments and revised the article. CY carried out most of the animal and cell experiments and wrote the manuscript. WG and XL conducted parts of cell experiments, such as plasmid transfection and lentivirus vector transduction. QW analyzed the experimental results and developed analysis tools. LiZ and YL fed experimental animals and conducted parts of animal experiments.

## Conflict of Interest Statement

The authors declare that the research was conducted in the absence of any commercial or financial relationships that could be construed as a potential conflict of interest.
